# Establishment of Alternative Culture Method for Spermatogonial Stem Cells Using Knockout Serum Replacement

**DOI:** 10.1371/journal.pone.0077715

**Published:** 2013-10-28

**Authors:** Keisuke Aoshima, Ai Baba, Yoshinori Makino, Yuki Okada

**Affiliations:** 1 Laboratory of Pathology and Development, Institute of Molecular and Cellular Biosciences, The University of Tokyo, Bunkyo-ku, Tokyo, Japan; 2 Career-Path Promotion Unit for Young Life Scientists, Center for the Promotion of Interdisciplinary Education and Research, Kyoto University, Yoshida Konoe Cho, Sakyo-ku, Kyoto, Japan; 3 Division of Molecular Pathobiology, Center for Zoonosis Control, Hokkaido University, Kita-ku, Sapporo, Japan; 4 Laboratory of Bioimaging and Cell Signaling, Graduate School of Biostudies, Kyoto University, Yoshida Konoe Cho, Sakyo-ku, Kyoto, Japan; 5 PRESTO, Japan Science and Technology Agency (JST), Saitama, Japan; University of Nevada School of Medicine, United States of America

## Abstract

Since spermatogonial stem cells (SSCs) are capable of both self-renewal and differentiation to daughter cells for subsequent spermatogenesis, the development of an efficient *in vitro* culture system is essential for studies related to spermatogenesis. Although the currently available system is serum-free and contains only chemically-defined components, it highly relies upon bovine serum albumin (BSA), a component with batch-to-batch quality variations similar to those of fetal bovine serum. Thus, we searched for an alternative BSA-free culture system that preserved the properties of SSCs. In this study, we utilized Knockout Serum Replacement (KSR) in the SSC culture medium, as a substitute for BSA. The results demonstrated that KSR supported the continuous growth of SSCs *in vitro* and the SSC activity *in vivo* without BSA, in a feeder-cell combination with mouse embryonic fibroblasts. The addition of BSA to KSR further facilitated cell cycle progression, whereas a transplantation assay revealed that the addition of BSA did not affect the number of SSCs *in vivo*. The combination of KSR with BSA also allowed the elimination of GFRA1 and FGF2, and the reduction of the GDNF concentration from 20 ng/ml to 5 ng/ml, while maintaining the growth rate and the expression of SSC markers. Furthermore, KSR was also useful with SSCs from non-DBA/2 strains, such as C57BL/6 and ICR. These results suggested that KSR is an effective substitute for BSA for long-term *in vitro* cultures of SSCs. Therefore, this method is practical for various studies related to SSCs, including spermatogenesis and germ stem cell biology.

## Introduction

Spermatogonial stem cells (SSCs) are the most primitive male germ cells in adult individuals, and are responsible for constitutive sperm production throughout life. Similar to other types of adult stem cells, SSCs undergo either self-renewal or asymmetric cell division, with the latter producing daughter cells (i.e., differentiated spermatogonia). The choice between self-renewal or differentiation is profoundly regulated by both intrinsic and extrinsic factors. The extrinsic factors are quite complex, because SSCs *in vivo* are surrounded by various types of somatic cells and differentiated spermatogonia. For example, Sertoli cells exist in seminiferous tubules and support the growth of neighboring germ cells, both structurally and as a source of cytokines and hormones. Thus, it was difficult to identify the essential extrinsic factors for culturing SSCs *in vitro*, until Nagano *et al.* reported the first example of an *in vitro* culture method using feeder cells [1]. Subsequently, Kubota *et al*. generated chemically-defined culture conditions by the addition of 0.2% (w/v) bovine serum albumin (BSA), and identified certain extrinsic cytokines, including glial cell line-derived neurotrophic factor (GDNF), GDNF family receptor alpha 1 (GFRA1) and fibroblast growth factor 2 (FGF2) [2], [3]. This improvement allowed SSCs to be cultured for longer periods without altering their undifferentiated properties, thus providing an unlimited supply of SSCs. Therefore, this method has been utilized for various SSC studies, such as gene manipulation and molecular/biochemical experiments. Similarly, Kanatsu-Shinohara *et al*. also developed a distinct culture system for germ-line stem cells (GSCs) using StemPro34-SFM, a serum-free medium originally developed for human hematopoietic cell culture [4]. However, the culture of GSCs requires 1% (v/v) fetal bovine serum (FBS) as well as 0.5% (w/v) BSA and cytokines, including GDNF and FGF2 [4].

As mentioned above, these present culture methods strongly rely upon the quality of the BSA. Similar to the use of animal serum products, such as FBS, problems often arise because of inconsistencies due to variations between batches and manufacturers. Thus, the establishment of a completely defined culture medium for SSCs without animal products has been eagerly anticipated.

For this purpose, we investigated the utility of Knockout Serum Replacement (KSR), a commercially available, defined cell culture supplement, for culturing mouse SSCs, based on a recent report by Sato *et al.* [5]. In this report, KSR was successfully used for *in vitro* testicular organ culture without serum and cytokines. Interestingly, they also found that AlbuMAX, a lipid-rich, high-quality BSA, could be used as a substitute for KSR. These observations suggested that BSA could be replaced by KSR for culturing SSCs *in vitro*. We also examined the compatibility of KSR with SSCs isolated from various mouse strains, as well as the possibility of reducing the cytokine supplementation in the culture medium containing KSR.

## Materials and Methods

### Preparation of Feeder Cells

Mouse embryonic fibroblasts (MEFs) were isolated from ICR mouse embryos at embryonic day 13.5 (CLEA Co. Ltd., Japan). SIM mouse embryo-derived thioguanine- and ouabain-resistant fibroblasts (STOs) were purchased from the RIKEN BioResource Center Cell Bank (RCB0536). They were maintained in D-MEM (WAKO #044-29765) supplemented with 10% FBS (EQUITECH-BIO), GlutaMAX™-I (Gibco #32561-037) and penicillin/streptomycin (Gibco #15140-122). After 3 or 4 passages, they were treated with 10 µg/ml mitomycin C (Wako #134-0791) for 2 hours. After the treatment, the cells were washed twice with PBS and trypsinized, and then 5.0 or 2.5×10^4^ cells/well of MEFs and 7.5 or 3.75×10^4^ cells/well of STOs were placed in 0.2% gelatin-coated 24 well- or 48 well-plates, respectively.

### Culture Medium

The composition of the basal culture medium for SSCs was described by Kubota and Brinster [3]. Briefly, Minimum Essential Media alpha (MEMα) was supplemented with GlutaMAX™-I (Gibco #32561-037), penicillin/streptomycin (Gibco #15140-122), 2-mercaptoethanol (Gibco #21985-023), 5 µg/ml insulin (SIGMA #I5500), 10 µg/ml transferrin (SIGMA #T1283), 0.5 ml/L Fatty Acid Supplement (SIGMA # F7050), 7.6 ueq/L Free Fatty Acid mixture {0.4 µM Linolenic acid (SIGMA #L2376), 1.0 µM Oleic acid (SIGMA #O1008), 0.2 µM Palmitoleic acid (SIGMA #P9417), 2.7 µM Linoleic acid (SIGMA #L1012), 2.4 µM Palmitic acid (SIGMA #P0500) and 0.9 µM Stearic acid (SIGMA #S4751)}, 30 nM Sodium selenite (SIGMA #S1382), 10 mM HEPES (Dojin Kagaku # 342-01375), 60 µM Putrescine (SIGMA #P7505), 1 ng/ml FGF-Basic Human Recombinant (Peprotech #AF-100-18B), 20 ng/ml Human GDNF Unconjugated (Peprotech #450-10) and 150 ng/ml Recombinant Rat GFR alpha-1 Fc Chimera CF (R&D Systems #560-GR-100/CF). MEM non-essential amino acids solution (Gibco #11140-050) and MEM Vitamin solution (Gibco #11120-052) were also added. Depending on the experimental design, 0.2% (w/v) bovine serum albumin (MP Biochemicals #810661, Lot #1110J) and 2 or 10% (v/v) Knockout Serum Replacement (Gibco #10828-028) were added.

### Isolation of SSCs

SSCs were isolated from postnatal day 8 testes of DBA/2NCrlCrlj (Charles River Laboratories Japan, Inc.), Jcl: ICR (CLEA Japan, Inc.), C57BL/6JJcl (CLEA Japan, Inc.) and 129X1/SvJJmsSlc (Japan SLC, Inc.) mice, according to Kubota and Brinster [3]. Briefly, the testes were excised from the pups and washed with Hanks’ Balanced Salt Solution (HBSS) (Gibco #14170-161), after the tunica albuginea was removed. The testes were then digested by a solution of 5 mg/ml DNase I (SIGMA #DN25) and 0.25% Trypsin-EDTA (Gibco #15400), to disperse the seminiferous tubules. The reaction was stopped by adding fetal bovine serum (FBS, Biofill #FBS01-500 Lot #AB20106). The cells were filtered through a 35 µm pore-cell strainer (BD Falcon #352235) and were centrifuged (600 g, 4°C, 3 min). The pellets were resuspended in HBSS, and layered onto a Percoll solution, which was centrifuged without using the centrifuge brake (600 g, 4°C, 8 min). The pellet was washed with PBS supplemented with 1% FBS, 10 mM HEPES, 1 mg/ml glucose (SIGMA #G7020-1KG), and penicillin/streptomycin (PBS-S), and was centrifuged again (600 g, 4°C, 3 min). The pellets were resuspended in PBS-S. Magnetic microbeads conjugated with anti-CD90.2 (Miltenyi Biotech #120-000-295) were added to the cell suspensions, which were incubated at 4°C for 30 min. The cell suspensions were applied to an MS column (Miltenyi Biotech #130-042-201), and the magnetically retained cells were collected. After centrifugation at 600 g at 4°C for 3 min, the pellets were resuspended in culture medium, and spread onto feeder cells.

### SSC Transplantation

CD1-Foxn1nu (also known as ICR nude) male mice were used as recipients. The 5-week-old recipients were treated with busulfan (SIGMA, #B2635, 40 mg/kg i.p.) to deplete the endogenous germ cells. At 4–6 days after treatment, the mice were transplanted with bone marrow, to prevent pancytopenia caused by the busulfan treatment. Transplantation was performed at 5–6 weeks after busulfan treatment. SSCs were isolated from the P8 testes of DBA/2 mice carrying the histone H4-Venus gene, in which the H4-Venus fusion protein was expressed by the histone H4 promoter (Makino *et al*., unpublished) as described above. The isolated SSCs were cultured in medium containing 10% KSR and 0.2% BSA for 3–4 weeks, and subsequently with or without BSA for 3 weeks. Immediately before transplantation, the SSCs were trypsinized and the feeder cells were removed, using a kit (Miltenyi Biotech #120-007-444), to eliminate the MEFs. A total of 0.5–2.0×10^5^ SSCs were injected per testis (n = 3 per group). At 8 weeks after transplantation, the testes were removed from the recipient mice for further examination.

### Immunostaining of SSCs

After the culture medium was removed from the wells, the cells were washed with PBS, fixed with 4% paraformaldehyde (Wako #163-18435) for 20 min at room temperature (RT), and then washed with PBS three times. Permeabilization was performed with 0.1% Triton X-100 at RT for 10 min, followed by two washes with PBS. Blocking was performed with 1.5% Blocking reagent (Roche #1096176) at RT for 1 hour. After blocking, the cells were incubated with the Anti-GENA monoclonal antibody clone TRA98 (Bio Academia #73-003) (1∶500) at 4°C overnight, and Alexa Fluor 488 goat anti-Rat IgG (Molecular Probes #A11006) (1∶1,000) was used for detection.

### Feeder Cell Removal, RNA Extraction and RT-qPCR

The SSCs and feeder cells were detached from the culture plates by trypsinization. The feeder cells were then removed by using Feeder Cell Removal microbeads (Miltenyi Biotech #120-007-444), according to the manufacturer’s protocol. Total RNA was extracted using a NucleoSpin® RNA II kit (Macherey-Nagel/TaKaRa BIO #74095510). The total RNA from mouse J1 embryonic stem cells (ESCs, ATCC® SCRC-1010™) was kindly provided by Dr. T. Kawamura, of Kyoto University. Reverse transcription was performed by using the SuperScript®III First Strand Synthesis System (Invitrogen #18080-051), according to the manufacturer’s protocol. Quantitative PCR (qPCR) samples were prepared with KAPA SYBR FAST qPCR Kit Master Mix (2X), using an ABI Prism Genetic Analyzer (KAPA BIOSYSTEMS #KK4603). The reactions were performed with an ABI StepOne PCR system (Applied Biosystems). The PCR primers are listed in Table S1. The results were normalized to the expression level of ribosomal protein S7 (Rps7), because it was stable and independent of the differentiation state of the SSCs (data not shown).

### Cell Cycle Analysis by Flow Cytometry

The SSCs maintained by MEF_B10K were transferred to the culture conditions indicated in the figure, and then cultured for 5 days before analysis. The SSCs and feeder cells were detached from the culture plates by trypsinization, and suspended in PBS-S. The cells were incubated with Vybrant® DyeCycle™ Orange Stain (Life Technologies #35005) at 37°C for 30 min in the dark. They were filtered through a 35 µm pore-cell strainer (BD Falcon #352235) and subjected to FACS analysis with a BD Accuri C6 flow cytometer (Becton Dickinson). The cell cycle analysis was performed with the Flowjo software (Treestar, ver 7.6.5).

### Ethics Statement

All experimental procedures involving animals were approved by the Animal Experiment Ethics Committees at the Graduate School of Medicine, Kyoto University (MedKyo11094) and the Institute of Molecular and Cellular Biosciences, The University of Tokyo (#23015). The experiments were conducted in accordance with the Guidelines for the Care and Use of Laboratory Animals of Kyoto University and The University of Tokyo. All efforts were made to minimize animal suffering and discomfort and to reduce the number of animals used.

## Results

### SSCs Grow Efficiently in the Presence of KSR on MEFs

**Figure 1:**
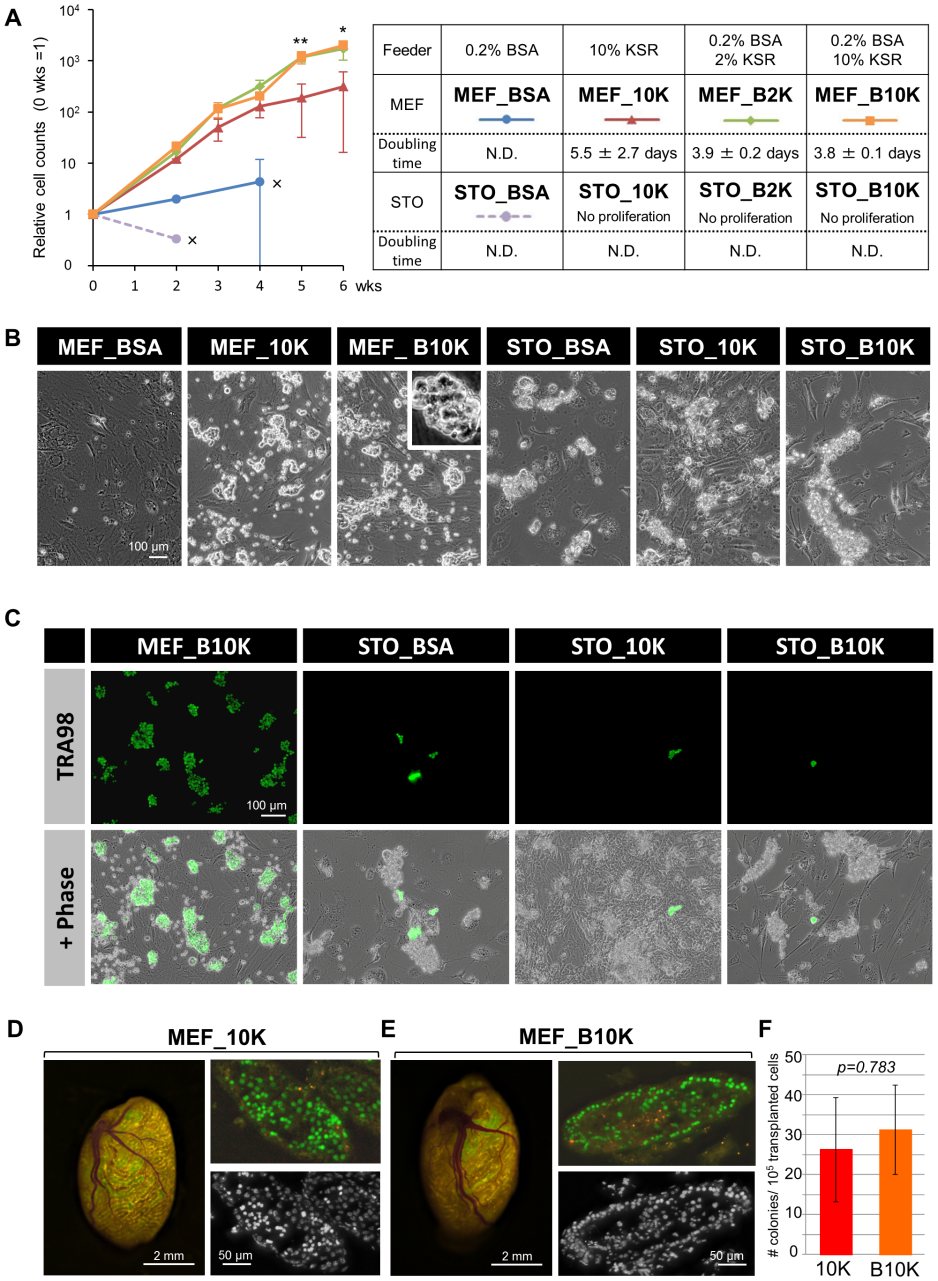
KSR can substitute for BSA in SSC cultures on MEFs. (A) Growth curves of SSCs cultured with the various combinations of BSA, KSR, MEFs and STOs (left panel) summarized in the table (right panel). Week zero indicates the day when the SSCs were isolated from postnatal day 8 testes of DBA/2 mice. The number of SSCs was counted at the indicated time points, and the cell counts are presented as means ± s.d. from three independent biological repeats There are statistically significant differences in the data from 5 and 6 wks between MEF_B2K/B10K and MEF_10K (*p<0.05, **p<0.01, ANOVA, Tukey’s HSD test). Crosses (×) with STO_BSA and MEF_BSA indicate that cells could not be counted because of massive cell death and exfoliation. No proliferation was observed in SSCs cultured under the STO_10K, STO_B2K and STO B10K conditions. The calculated doubling times are also shown in the table (right panel). N.D., not determined due to inefficient proliferation. (B) Phase contrast images of SSCs from the indicated culture conditions. Scale bar, 100 µm. The magnified view shows a “grape-shaped” colony, which is typical for undifferentiated SSCs. (C) Immunofluorescent analysis of SSCs, grown under different culture conditions, with the anti-GENA antibody clone TRA98, a germ cell-specific marker. TRA98-positive cells are green in the upper panels, and phase contrast-merged images are shown in the lower panels. Scale bar, 100 µm. (D–E) Representative images of mouse testes transplanted with Venus-expressed MEF_10K (D) or MEF_B10K SSCs (E), respectively. Gross Venus fluorescence images (left panels) and histological sections (Venus fluorescent images in upper panels, and Hoechst images in lower panels) are shown. Donor-derived Venus-positive cells are green, and the yellow fluorescence is caused by the auto-fluorescence of testicular tissue. Scale bars are as indicated. (F) Quantification of stem cell activities of MEF_10K and MEF_B10K by a transplantation assay. The graph presents the number of Venus-positive SSC colonies per 10^5^ transplanted cells ± S.E.M (n = 3 per group). There was no significant difference between MEF_10K and MEF_B10K by Student *T* test (*p = 0.783*).

To examine the effect of KSR on the SSC culture, we tested four different combinations of BSA and KSR, as summarized in Fig. 1A. In addition, the compatibility with two types of popular feeder cells (STOs and MEFs) was also examined (Fig. 1A). Among these conditions, 0.2% BSA and STOs (STO_BSA) corresponded to the conventional conditions reported by Kubota *et al.* [2]. After 6 weeks of culture on MEF feeder cells, a substantial cell growth was observed with 10% KSR alone (MEF_10K). The doubling time was 5.5±2.7 days, which was equivalent to that of the conventional culture method (5.6±0.2 days, [2]) (Fig. 1A). Remarkably, SSCs cultured with 0.2% BSA plus 2 or 10% KSR (MEF_B2K and MEF_B10K) exhibited the highest growth rates (the doubling times were 3.9±0.2 days for MEF_B2K and 3.8±0.1 days for MEF_B10K) as compared to the MEF_10K and other conditions (Fig. 1A–B). To the contrary, BSA alone (MEF_BSA) failed to support the cell growth, as most of the cells detached from the feeder cells before they formed colonies (Fig. 1A–B). These data suggested that KSR alone can maintain the *in vitro* growth of SSCs by substituting for BSA, when MEF cells were used as feeder cells. Furthermore, the addition of BSA to KSR significantly accelerated the cell growth, even though by itself it is incapable of supporting cell growth (Fig. 1A–B). In contrast, in the case of using STO feeder cells, only STO_BSA exhibited transient colony formation, which remained small, and eventually disappeared within 2 weeks (Fig. 1A–B). No proliferation of SSCs was observed in STO_10K, STO_B2K and STO_B10K after the culture was initiated, indicating that STO failed to support the growth of SSCs, even in the presence of both KSR and BSA (Fig. 1A–B).

Morphologically, MEF_B10K SSCs formed “grape-shaped” colonies, which were typical for SSC colonies (Fig. 1B, inset). These colonies were all positive for αGENA TRA98, a specific marker of germ cells, whereas the numerous cell clumps observed in cultures with STOs contained very few αGENA TRA98 positive cells, indicating that they were composed of either dead germ cells or originated from the STOs (STO_BSA, STO_10K, and STO_B10K, Fig. 1C).

Finally, we tested whether SSCs cultured with KSR maintained the stemness *in vivo*. Since previous studies revealed that the *in vitro* cultured cells usually consist of SSCs and non-stem cell progenitors, with the latter having lost their self-renewal capability [6], the MEF_BSA, MEF_10K and MEF_B10K SSCs were subject to transplantation into the testes of busulfan-treated ICR nude mice, in which their own germ cells were depleted. Unfortunately, however, MEF_BSA could not be tested, because we were unable to obtain a sufficient number of cells for the transplantation, due to poor cell growth (Fig. 1A). Unexpectedly, although MEF_B10K allowed the cells to proliferate significantly faster than MEF_10K *in vitro* (Fig. 1A), both MEF_10K and MEF_B10K cells were successfully engrafted with equivalent stem cell activity (Fig. 1D–F), strongly indicating that KSR alone is capable of maintaining the SSC activity *in vivo*.

### Gene Expression Properties are Maintained in SSCs Cultured with KSR

**Figure 2:**
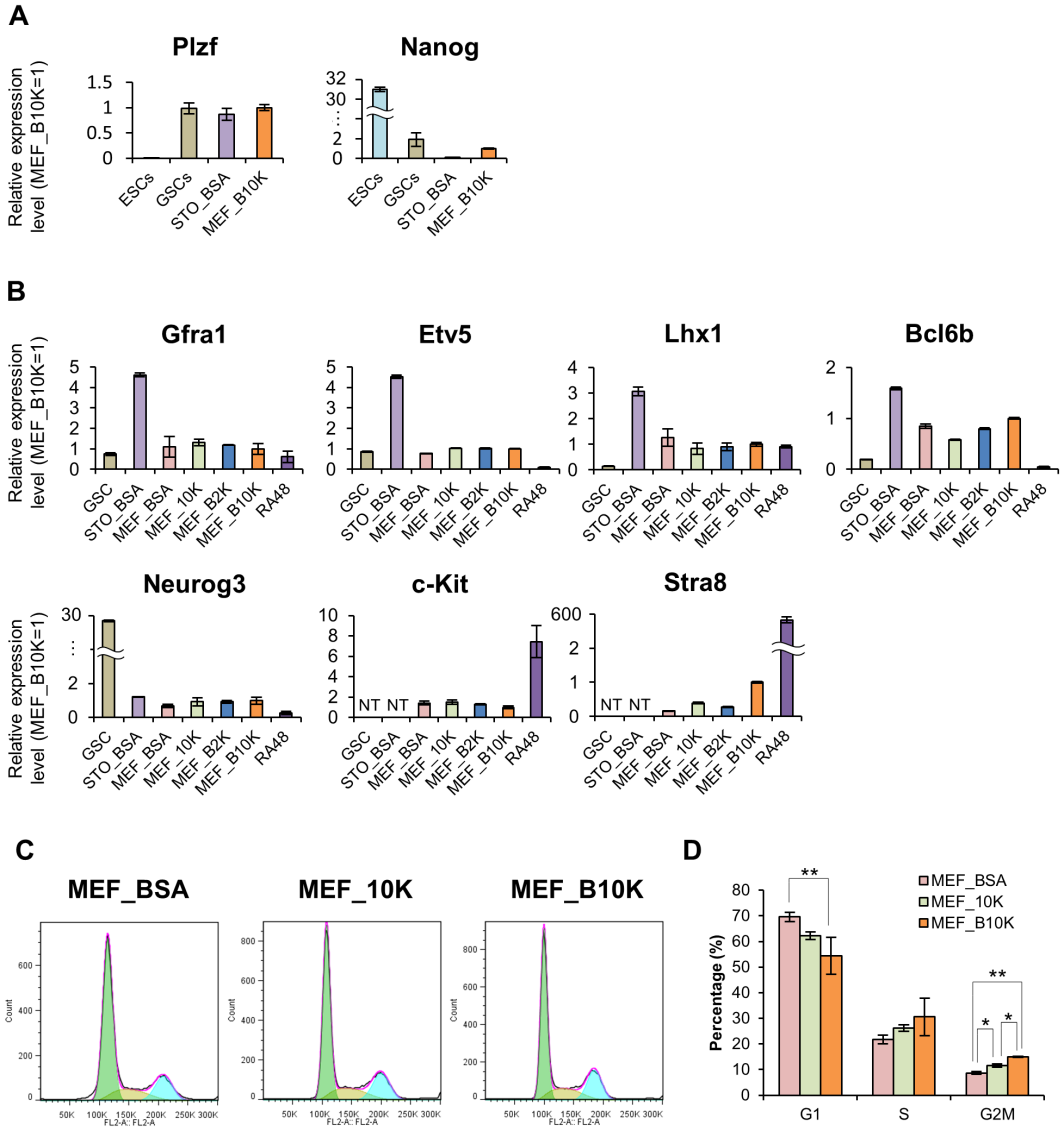
Gene expression properties are maintained in SSCs cultured with KSR. (A) Relative expression levels of marker genes for undifferentiated spermatogonia (Plzf) and ESCs (Nanog). Data are presented as means ± s.d. from three independent experiments. The Y-axis indicates the relative expression level, with the value of MEF_B10K set to 1 for each group. (B) Relative expression levels of other undifferentiated spermatogonial markers (Gfra1, Etv5, Lhx1, Bcl6b, and Neurog3) and differentiated spermatogonial markers (c-Kit and Stra8). Data are presented as means ± s.d. from three independent experiments. The Y-axis indicates the relative expression level, with the value of MEF_B10K set to 1 for each group. (C) Cell cycle analyses by flow cytometry at 5 days after the medium change from MEF_B10K to each indicated condition. Histograms are representative examples of each sample. The tables under each histogram indicate the percentages of each cell cycle stage. The histograms are colored according to each cell cycle stage. Green: G1 phase. Ocher: S phase. Light blue: G2/M phase. (D) Quantification of the results shown in (C). The graph is presented as means of the percentages ± s.d. from three independent experiments. Asterisks indicate statistical significance (*p<0.05, **p<0.01, ANOVA, Tukey’s HSD test).

Since we had confirmed that the SSCs cultured with KSR possess stemness *in vivo*, we next performed RT-qPCR to investigate whether the gene expression properties of SSCs were influenced by KSR. Since KSR was originally developed to maintain ESCs, we first examined the expression levels of Nanog and PLZF. Nanog is highly expressed in ESCs as well as primordial germ cells, but is disappeared in male germ cells by E16.5 [7], whereas PLZF is a marker of undifferentiated spermatogonia, including SSCs [8]. For this purpose, we compared MEF_B10K with STO_BSA, a previously reported conventional condition, and GSCs, which are also considered to be SSCs originated from gonocytes existing only in infant testis, and can be cultured *in vitro* in a distinct culture medium with a doubling time of approximately 2.6 days [4]. Mouse ESCs served as positive and negative controls for Nanog and PLZF, respectively. The results demonstrated that MEF_B10K produced higher levels of Nanog, as compared with STO_BSA, but it was comparable to GSCs (Fig. 2A). The expression level of Plzf was similar among the three samples (Fig. 2A), indicating that the cells cultured with MEF_B10K properly maintained the status of undifferentiated spermatogonia.

We next compared the mRNA expression levels in cells cultured on MEFs with different combinations of KSR and BSA, as shown in Fig. 1A, to see if the gene expression patterns were related to the growth rates. To evaluate the differentiation status, five markers for undifferentiated spematogonia (GFRa1, Etv5, Bcl6b, Lhx1, and Neurog3) and two markers for differentiating/differentiated spermatogonia (c-Kit and Stra8) were tested. The cells in each group were prepared by changing the media from MEF_B10K to either MEF_BSA, MEF_10K, or MEF_B2K, and then culturing them for three weeks before isolating the RNA. However, the cells in the MEF_BSA culture started to detach from the feeder cells a few days after the medium was changed, as we observed in Fig. 1A. Therefore, we stopped that culture at day 10, and isolated the RNA. As a result, all four samples cultured on MEF (i.e., MEF_BSA, MEF_10K, MEF_B2K and MEF_B10K) exhibited intermediate expression levels of undifferentiated spematogonial markers, between those of GSCs and STO_BSA [9] (Fig. 2B). However, no crucial differences in the expression levels of these genes were observed among the four samples, although the proliferation of MEF_BSA was quite limited. Similarly, the expression levels of c-Kit and Stra8, which are markers of differentiating/differentiated spermatogonia, were also unaltered, except for the subtle increase of Stra8 in MEF_B10K (Fig. 2B, Fig. S1). Taken together, these results suggested that utilizing KSR is effective to ensure the cell proliferation *in vitro,* without altering the gene expression properties.

To further investigate how KSR facilitated the cell growth, we next examined the effect of KSR on the cell cycle. At 5 days after changing the culture conditions from MEF_B10K to either MEF_BSA or MEF_10K, we observed a significant decrease of cells in G2/M phase in MEF_BSA accompanied by a marked increase of cells in G1-phase and a subtle reduction of cells in S-phase (Fig. 2C). MEF_10K exhibited the intermediate phenotype between MEF_B10K and MEF_BSA in each phase of cell cycle (Fig. 2C), clearly suggesting that KSR can ensure the cell cycle progression, and the combined use of KSR with BSA further supports the effect on the cell cycle *in vitro*.

### The Addition of GDNF is Sufficient in the Presence of KSR

**Figure 3:**
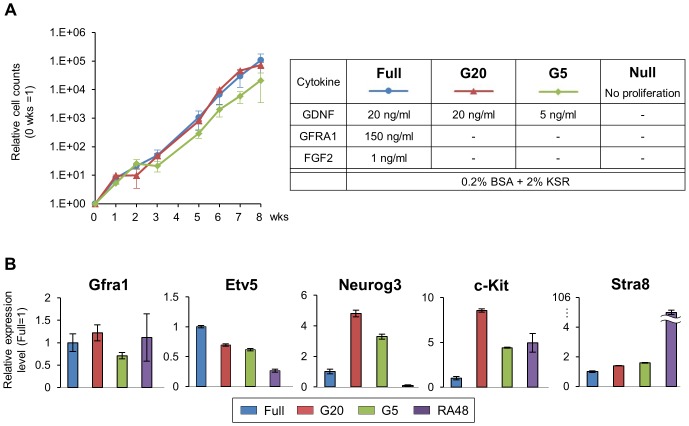
GDNF is indispensable but can be reduced in the presence of KSR. (A) Growth curves of SSCs under reduced cytokine conditions (left panel), summarized in the table (right panel). The number of SSCs was counted at the indicated weeks, and the cell counts are presented as means ± s.d. from three independent experiments. No proliferation was observed in Null condition. The calculated doubling times are also shown in the table (right panel). N.D., not determined due to inefficient proliferation. (B) Relative mRNA expression levels of undifferentiated spermatogonial markers (Gfra1, Etv5and Neurog3) and differentiating/differentiated spermatogonial markers (c-Kit and Stra8) were examined at 12 hours after the medium change from the full cytokine conditions to the indicated conditions. RA48 cells are differentiated SSCs treated with retinoic acid for 48 hours. Data are presented as means ± s.d. from three independent experiments. The Y-axis indicates relative expression level to the value of Full, set to 1 for each group.

The presence of certain cytokines is essential to maintain the stemness of SSCs *in vitro* by creating a favorable environment, and several cytokines and growth factors that enhance the proliferation of SSCs have been identified. Among them, GDNF, provided by the Sertoli cells adjacent to the SSCs *in vivo,* is crucial for the self-renewal of SSCs *in vitro* as well [2]. In addition to GDNF, GFRA1 and FGF2 also support the continuous proliferation of SSCs *in vitro*. Since the combination of KSR and BSA (MEF_B2K or MEF_B10K) significantly facilitated the cell proliferation *in vitro*, we next tested whether the presence of both KSR and BSA could allow the variety and/or amounts of cytokines to be reduced. Since MEF_B2K and MEF_B10K exhibited similar growth rates (Fig. 1A), MEF_B2K was used as the basal medium, and four distinct culture conditions with reduced amounts/types of cytokines, as shown in Fig. 3A, were tested to assess the effects on cell growth and gene expression.

The cell growth analysis revealed that the removal of GFRA1 and FGF2 did not affect the proliferation of SSCs as long as GDNF was supplemented, suggesting that these two cytokines were dispensable in the presence of KSR (Fig. 3A). Furthermore, although the complete elimination of GDNF caused severe growth retardation, a 75% reduction in the GDNF concentration (from 20 ng/ml to 5 ng/ml) still supported continuous cell proliferation, with doubling times of 3.5±0.1 days (20 ng/ml GDNF) and 4.0±0.3 days (5 ng/ml GDNF) (Fig. 3A). The gene expression analysis revealed that the expression levels of undifferentiated markers (Etv5, Gfra1) were maintained even when the amounts of the cytokines were reduced (Fig. 3B). On the other hand, the expression levels of Neurog3 and c-Kit were greatly increased without GFRA1 and FGF2, but the cell growth was unaltered. However, although the increase in c-Kit indicated that the cells were differentiating, the expression of Stra8, a marker of further differentiated spermatogonia, remained low, indicating that the SSCs without GFRA1 and FGF2 were not completely differentiated, and thus were capable of proliferation *in vitro*. These results demonstrated that KSR is effective to maintain cell growth in the presence of lower cytokine concentrations in the SSC culture medium.

### SSCs Derived from ICR and B6 Mice can be Cultured Under the Alternative Conditions

**Figure 4:**
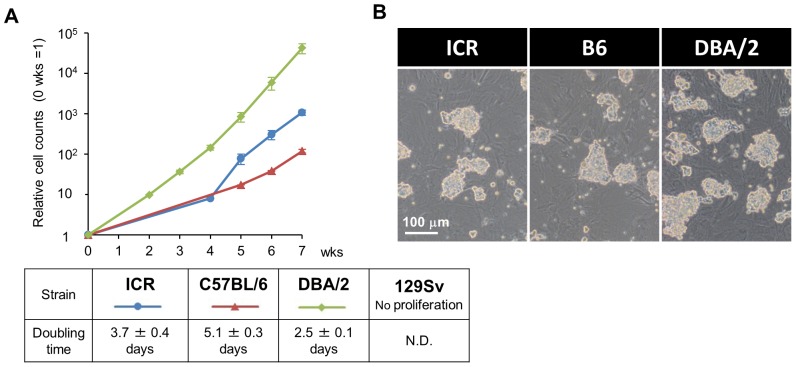
SSCs derived from ICR and B6 can be cultured under the alternative conditions. (A) Growth curves of SSCs derived from ICR, B6 and DBA/2 mice (left panel), summarized in the table (right panel). The number of SSCs was counted at the indicated time points, and cell counts are presented as means ± s.d. from three independent experiments. The calculated doubling times are also shown in the table (right panel). (B) Phase contrast images of SSC colonies derived from ICR, B6 and DBA/2 mice. Scale bar, 100 µm.

One of the most influential factors for culturing SSCs *in vitro* is the mouse strain from which the SSCs are isolated. Previous studies demonstrated that SSCs isolated from DBA/2 mice proliferate more quickly than those from other strains such as C57BL/6 (B6), which reportedly requires GFRA1 and FGF2 for continuous proliferation [2], [3]. In fact, presumably due to a substandard batch of BSA for the SSC culture, we were unable to culture SSCs from non-DBA/2 strains *in vitro* under the conventional culture conditions, despite multiple attempts and increased concentrations of cytokines (data not shown). Thus, we next tested whether the combination of KSR and BSA was applicable to SSCs from non-DBA/2 strains, including B6, ICR and 129 Sv. For this experiment, we used all of the recommended cytokines (i.e. GDNF, GFRA1, and FGF2), because a previous study demonstrated that SSCs isolated from B6×B6∶129 (i.e. 75% B6 and 25% 129 Sv) mice require these cytokines for efficient proliferation [2]. As a result, although more time was required for the initiation of the proliferation, the SSCs derived from the B6 and ICR strains started to proliferate exponentially after about 4 weeks, and the doubling times between the 5^th^ and the 7^th^ week were calculated as 3.7±0.4 days (ICR), 5.1±0.3 days (C57BL/6) and 2.5±0.1 days (DBA/2) (Fig. 4A). Once they started to proliferate regularly, they formed tight colonies, which were indistinguishable from those of DBA/2 (Fig. 4B), implying the effectiveness of KSR for non-DBA/2 strains. However, the SSCs from 129 Sv, which are reportedly the most difficult to establish, never proliferated under this condition, and thus further modifications are required for 129 Sv.

## Discussion

For cell culture, the development of serum-free, defined culture medium is beneficial to disseminate experimental methods to other laboratories with high reproducibility and reliability, since the quality of serum varies depending on the batch, the manufacturers’ preparation methods, and even between individual animals. In addition, primary cells are usually sensitive to serum-containing inhibitory materials, whereas fibroblast-derived feeder cells selectively proliferate due to the growth factors within FBS. One of the major purposes for adding serum is to provide hormones or growth factors to stimulate cell replication. Therefore, supplementation of the media with optimal amounts of individual hormones and growth factors can eliminate the need for serum, without affecting the growth or other characteristics of the cells. For SSCs, previous studies reported the development of defined serum-free media by utilizing ideal cytokines, which support the long-term culture while maintaining the stemness [2], [3]. However, these reported conditions are strongly dependent on the BSA, which sometimes causes spontaneous differentiation of SSCs due to its inconsistent quality as an animal product.

In this study, we demonstrated that KSR, a commercially available serum-free supplement, can substitute for the BSA in both *in vitro and in vivo* SSC cultures without affecting their properties, such as the stem cell activity and gene expression. In addition, the combined use of KSR with BSA significantly ensures cell proliferation by facilitating cell cycle progression. KSR was initially employed for ESCs, since it prevented spontaneous differentiation, and it was subsequently utilized for other types of stem cells including human embryonic germ cells [10], suggesting its usefulness for culturing SSCs. In support of this idea, Sato *et al*. recently demonstrated that KSR is vital for testicular organ culture, and they further verified that KSR and high-quality BSA exert similar effects, suggesting that KSR is useful as an alternative for BSA for *in vitro* culture of SSCs [5]. However, another group reported that KSR did not support the long-term culture of SSCs, when it was used with DMEM/F12 basal medium and STO feeders [11], suggesting that compatibility with the basal medium and feeder cells is also critical. Indeed, we were unable to utilize KSR along with STO feeder cells and the conventional MEMα-based medium previously reported by Kubota *et al*. [2], [3], and therefore MEF feeders were used instead (Fig. 1A). Since the conventional medium is reportedly compatible with STOs, KSR might have a detrimental effect on STOs, as we observed morphological alterations of the STOs in the presence of KSR (Fig. 1B). Similarly, BSA alone was incompatible with MEFs in the absence of KSR in our culture conditions, as it caused the detachment of SSCs from MEFs (MEF_BSA, Fig. 1A). This was probably one of the major reasons why cell growth was inhibited, besides the impairment of cell cycle progression (Fig. 2C). Furthermore, it is also intriguing that KSR facilitates the cell cycle progression of SSCs, because this effect hasn’t been reported in ESCs and other types of stem cells.

Another remarkable finding is that even though KSR increased the growth rates of SSCs to a level close that of GSCs, the gene expression properties were still relatively similar to those of SSCs cultured under the conventional conditions. GSCs are isolated from gonocytes, which are more primitive germ cells than SSCs, and they are sometimes converted to ESC-like pluripotent stem cells that generate embryonic carcinomas [9]. This cancerous conversion would be disadvantageous for clinical usage, such as SSC transplantation for the treatment of infertility. From this viewpoint, the qualities of KSR, which enhances the cell growth and maintains the stem cell activity simultaneously, seem to be suitable for the clinical applications of SSCs.

In addition, KSR not only allowed a significant reduction in the GDNF concentration for SSCs from the DBA/2 strain, but also was effective for SSCs from non-DBA/2 strains, except for 129 Sv. Among the various mouse strains, B6 is the most widely-used strain for multiple purposes, while ICR is also popular because of its fecundity. Thus, as long as KSR is used, backcrossing to DBA/2 is no longer required, when these two mouse strains are used to isolate and culture the SSCs. Unfortunately, however, KSR failed to support the culture of 129 Sv SSCs, which are regularly used for *in vivo* gene targeting due to their high frequency of recombination. The exact reason for the strain-dependency is still unknown, but previous reports indicated that it might be caused by the sensitivity and/or dependency of the GNDF receptors. Thus, the effect of KSR is probably not due to an alteration of the GDNF pathway but to other reasons, such as osmolarity [12].

We concluded that KSR is a reliable substitute for BSA that does not affect the properties of SSCs. This finding provides efficient and stable culture conditions for SSCs and bypasses the problems with the use of BSA, and thus will facilitate various studies using SSCs, including therapeutic applications.

## Supporting Information

Figure S1 **Gene expression properties are maintained in SSCs cultured with KSR.** Relative expression levels of undifferentiated spermatogonial markers (Gfra1, Etv5, Lhx1, Bcl6b, and Neurog3) and differentiated spermatogonial makers (c-Kit and Stra8), measured 10 days after the shift to each condition from MEF_B10K. RA48 cells are differentiated SSCs treated with retinoic acid for 48 hours. Data are presented as means ± s.d. from three independent experiments. The Y-axis indicates the relative expression level, with the value of MEF_B10K set to 1 for each group.
(TIF)

Table S1 **Primer sequences for RT-qPCR.**
(DOC)
