# Supplementary figures and images for: Establishment of Alternative Culture Method for Spermatogonial Stem Cells Using Knockout Serum Replacement
Source: PLoS One. 2013 Oct 28;8(10):e77715. doi: 10.1371/journal.pone.0077715 (PMC3810131; doi:10.1371/journal.pone.0077715)

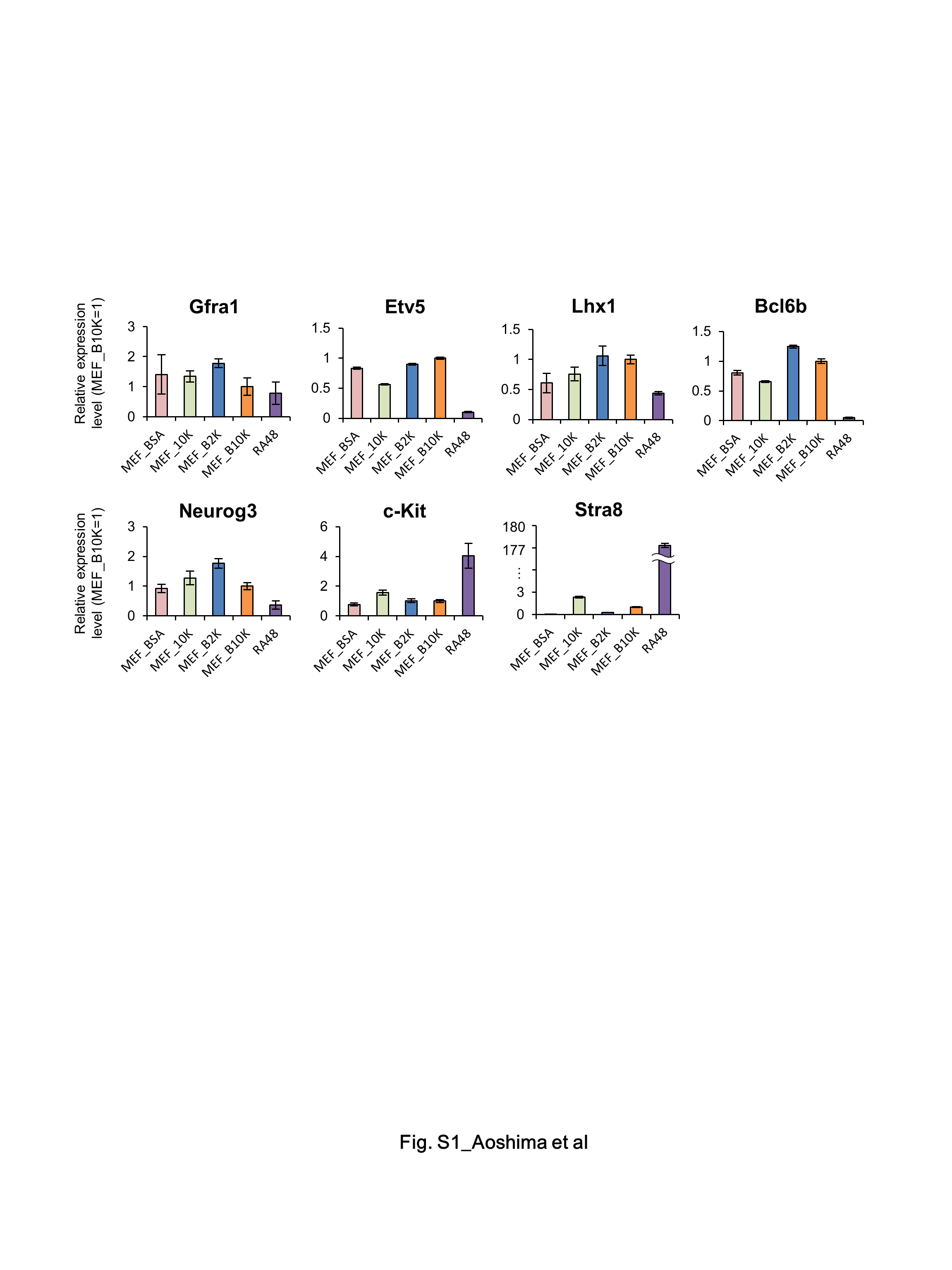

Supplement: Figure S1 — Gene expression properties are maintained in SSCs cultured with KSR. Relative expression levels of undifferentiated spermatogonial markers (Gfra1, Etv5, Lhx1, Bcl6b, and Neurog3) and differentiated spermatogonial makers (c-Kit and Stra8), measured 10 days after the shift to each condition from MEF_B10K. RA48 cells are differentiated SSCs treated with retinoic acid for 48 hours. Data are presented as means ± s.d. from three independent experiments. The Y-axis indicates the relative expression level, with the value of MEF_B10K set to 1 for each group. (TIF) [file pone.0077715.s001.tif]
