# Supplementary material for: Establishment of Alternative Culture Method for Spermatogonial Stem Cells Using Knockout Serum Replacement
Source: PLoS One. 2013 Oct 28;8(10):e77715. doi: 10.1371/journal.pone.0077715 (PMC3810131; doi:10.1371/journal.pone.0077715)
Supplement: Table S1 — Primer sequences for RT-qPCR. (DOC) [file pone.0077715.s002.doc]

| **Table. S1 Primer sequences for RT-qPCR** | | |
| --- | --- | --- |
| Primer | Sequence | |
|  | Forward | Reverse |
| Etv5 | GATCAGCAAGTCCCTTTTATGG | AAATCTGGGACAAACTGCTCAT |
| Gfra1 | CAGGGAAATGACCTACTGGAAG | TTGTTCCCTTTGGAAATGTGT |
| Ngn3 | AGACTCCCATACTTCCTGGTGA | AAGACGCAACACTGGATTAGGT |
| Bcl6b | CGTGCTTAGCAATCTGAATGAG | TGAATAGAAGAAGCCACTGCAA |
| Lhx1 | AGCGAAGGATGAAACAGCTAAG | GGGACCGTAGTACTCACTCTGG |
| Kit | TGACAAATTCACCCTCAAAGTG | GGAGTTCACGGATGTAGACACA |
| Stra8 | TGGATAAGTTGCTGAAGCTCAA | GGGACTGTCCTGAAGAAAACTG |
| Rps7 | AAAGCCGTACGAAAAATAAGCA | GTTTCACACGGATCCTCTTACC |
